# Supplementary material for: Head direction cells use a head-referenced dual-axis updating rule in 3D space
Source: Commun Biol. 2026 May 5;9:1012. doi: 10.1038/s42003-026-10086-2 (PMC13400642; doi:10.1038/s42003-026-10086-2)
Supplement: Supplementary file 3 — Transparent Peer Review file [file 42003_2026_10086_MOESM3_ESM.pdf]

nature portfolio

Peer Review File

Head direction cells use a head-referenced dual-axis updating rule in 3D space

Corresponding Author: Professor Kate Jeffery

This file contains all reviewer reports in order by version, followed by all author rebuttals in order by version.

Version 0:

Reviewer comments:

Reviewer #1

(Remarks to the Author)
In this manuscript, the authors build on the lab's previous studies demonstrating that Head Direction Cells (HDCs) use a so-called "dual-axis" (DA) updating rule to track head orientation during motion in 3D. This rule accumulates head rotations both in yaw (i.e. in the egocentric head-horizontal plane; HA) and around the allocentric earth-gravity axis (GA).

Here, the authors test an alternative hypothesis, namely that HDCs might be referenced to the locomotion surface plane (SA) as well as to the GA. Testing this hypothesis makes sense, since it seems plausible that HDCs might track motion relative to the surface on which the animal is navigating, and ignore 3D head movements outside of that surface's plane.

The authors record a comprehensive dataset of 436 cells and report a clear and unambiguous preference for a dual-axis updating rule referenced to the head axis (HA). The data analysis is rigorous and follows state-of-the-art methods. Overall, this is a methodologically robust, highly novel, and convincing study that addresses an important question about the information encoded by HDCs during natural 3D motion. The findings are highly relevant to the broader field of spatial navigation.

I have mainly minor remarks concerning clarity and interpretation of some results.

- Description of the GA rule (line 78): The description of the GA rule in line 78 is unclear and potentially inconsistent with the definition in line 414. Based on the definition in line 78, head rotation around an axis perpendicular to gravity would not update HDC. Yet, consider the following example: first, imagine that the head is vertical and define a vector u aligned with the nose. Second, pitch the nose up while keeping the vector u fixed in an earth reference frame. Third, rotate the head around u. Since u is orthogonal to the gravity axis, α would not be updated according to the definition in line 78. On the other hand, according to the definition in line 414, α would vary since the projection of the nose vector onto the earth-horizontal plane would change.

- Figure 3: For clarity, does the R value correspond to the correlation between the experimentally measured firing rate of the cell (as a function of time) and the firing rate predicted by the respective model, as defined in line 410?

- Equation 2: The authors seem to imply that αHH and αHG are two variables represented independently in the brain. Is this what they actually mean? Consider the following examples:
(1) Starting from the upright head position (both $\alpha = 0$), the head rotates 360° in yaw. This corresponds to $\alpha HH = 360°$ and $\alpha HG = 0$.
(2) Starting from the same position, the head pitches up until the nose is vertical, then rotates 360° around the gravity axis, and finally pitches back to horizontal. This corresponds to $\alpha HH = 0$ and $\alpha HG = 360°$.
In both cases, the initial and final head orientations are identical. However, if αHH and αHG are represented separately by the HD system, then the overall neuronal activity at the final position would differ between the two trajectories. What is the authors' interpretation of this? Note that I am not questioning the validity of the dual updating rule itself, but rather whether the HDC system represents two distinct forms of "azimuth."

- Line 222: Related to the point above, the authors write that "the added role of GA azimuth requires extension of standard ring attractor models." Alternatively, one could propose that HA and GA rotation velocities are computed upstream of the ring

attractor and that the sum of these signals is provided to the attractor. This would comply with the dual-axis updating rule without requiring an extension of the standard model.

- Line 175: As a side result, the authors report no tuning to elevation. However, the range of elevation sampled in their study appears rather narrow (Fig. S5). It is conceivable that some tuning to elevation (or other forms of 3D tuning) exists but cannot be observed given the limited elevation range sampled. I would encourage the authors to take that limitation into account.

Reviewer #2

(Remarks to the Author)
In this study, Williams et al. performed tetrode recordings from head direction (HD) cells in the anterodorsal thalamus (ADN) or postsubiculum (PoS) of rats as they foraged for malt paste on the top/sides of a mesh-covered 1-m diameter partial sphere to determine the rotational reference frame used by HD cells to maintain a coherent directional signal. Previous studies of HD cells during 3D locomotion have indicated that HD cells follow a “dual-axis” (DA) or “tilted azimuth” rule, such that HD cell firing is determined by both head rotations about a local axis as well as rotations of that local axis around the gravity axis. While previous studies have indicated definitively that HD cells follow this DA rule instead of some other single-axis rule (e.g., local axis only), here the authors use a modeling approach to assess if the DA axes are referenced to the orientation of the animal’s head itself (egocentric; DAH model) or the orientation of the local plane of locomotion (allocentric; DAS model), and compare these to a model based on the horizontal axis/gravity axis alone (GA model) as well as a head axis only model (HA model). Results indicate fairly convincingly that the DAH model provides the best fit for most cells.

This manuscript addresses the very important question of which reference frames are used by the brain to process spatial orientation in a 3D world. However, while the experiment is interesting and answers an important question about HD cell reference axes, my main criticism is that the paper is underdeveloped with regard to motivating and explaining the tested hypotheses, as well as placing the results into a broader context.

Major points

1. The main question of whether the local axis is referenced to the environment surface or the head itself is important, but not clearly motivated by the introductory text, other than mentioning that previous studies “did not address” it (line 66) and that “either is plausible” (line 67). Are there any reasons to expect one hypothesis over the other (DAH or DAS)? Does anything from the cited previous studies (or others – see point 3) indicate that one local axis is more likely? As HD cells have been demonstrated to rely on vestibular inputs, is there reason to believe they wouldn’t be referenced to the animal’s head itself (which already contains vestibular organs that detect head turns and gravity orientation)? Is there a potential biological basis for HD cells being somehow referenced to the plane tangent to the local surface? More thorough motivation of the experiment and reasoning behind each hypothesis is needed.

2. Related to point 1, there is also very little discussion of the results and their implications for spatial coding in the brain as well as behavior/navigation. For example, the authors conclude the main text by stating “How the brain processes these reference frames, and how and where they interact, remains to be elucidated” (Line 223-225). But there has been a sizable amount of experimental and theoretical work devoted to answering this question (see Laurens and Angelaki 2018 for review), and at least some of this work should be discussed to properly contextualize the results of the current study. Additionally, the authors should mention if there are any implications of their results for spatial coding downstream of the HD circuit. For example, entorhinal grid cells and hippocampal place cells are typically considered to represent allocentric locations on a particular surface. Further, grid cells have been shown to depend on anterior thalamus functon(Winter et al., 2015) and place cells and HD cells are strongly coupled in their orientation preferences (Knierim et al., 1995), suggesting that these systems interact. Since the current study shows that HD cell reference axis is egocentric, is there some transformation that needs to occur for the egocentric HD signal to properly interact with downstream allocentric grid and place signals (among other allocentric cell types)? Would the distinction between surface-referenced and head-referenced tuning in the ADN/PoS be expected to have specific effects on downstream neuronal firing properties, e.g. entorhinal or hippocampal cells, or on behavior in general? Overall a dedicated Discussion section would be welcome.

3. Mentions of several directly relevant studies are missing; for example, the paper mentions (Page et al., 2018) and (Laurens and Angelaki 2018) as recent evidence for the DA rule, but misses (Angelaki et al., Nat Comms, 2020), which showed evidence for the DA rule in mice on an orientable platform, and (LaChance et al., J Neurosci, 2020), which showed evidence for the DA rule in rats climbing on different faces of a cube. The (Angelaki et al., 2020) study is particularly relevant to the discussion of elevation tuning, which was observed in that study but not in the current study, which should be discussed more thoroughly. Many of the concepts critical to this paper were also explored in (Shinder and Taube, J Neurophys, 2018) which investigated HD tuning in head-fixed rats subjected to a variety of passive 3D rotations, and also concluded that a dual-axis rule was responsible for HD cell firing preferences (though they suggested that the local axis was primarily visual). The Shinder and Taube study may also be relevant because HD cells fired normally during passive 3D rotations despite the lack of a traditionally defined ‘local surface’ (the animals were head-fixed inside a tube).

Minor points
1. Line 32 – “exploring a hemisphere”, and Line 70 – “exploring a hemispherical arena” – need make clearer that the animals are crawling on the surface of the hemisphere instead of moving inside it (unlike the Grieves lattice maze, for example)
2. Line 50-51 – “However, HD cells do maintain a stable PFD on a sphere (Fig. 1b)….” The authors cannot make this definitive statement based solely on a single path/spike plot from one HD cell, they need to demonstrate it statistically or at

least show more examples
3. Fig. 1b – why does the side view only show a path with no cell firing, while the top views show cell firing but don't include a path alone? It could also be useful to see some occupancy distributions for locations on the top/sides of the sphere, since it seems like the animals rarely walked on the steep sides of the sphere (where the distinctions between the different models should be greatest)
4. Related to above point, the authors should include more examples of these path/spike plots for different cells so readers can better assess behavior and cell firing
5. Fig. 1d – axis labels are overlapping which makes them hard to read and interpret
6. Fig. 1e – the GA and DAH examples are clearly the same cell as mentioned in the legend (same vector locations), but why does the DAS example appear to be from a different cell/recording?
7. Fig. 1f – it would be useful to see the actual divergence between local PFDs and global PFD at different locations on the sphere (e.g., what is the degree or radian difference represented by the hottest color?). Do the actual HD cells show differences of a similar pattern/magnitude, either individually or as a population (an empirical version of this theoretical plot would be nice to see)? This may not be possible given the limited sampling on the sides of the sphere.
8. The authors should include more tuning curve examples in addition to the one shown in Fig. 2a. There are 40 example HA cells in Supplementary Fig. 6 which is the least common cell type, so they should be able to show more examples of the more common cell types (DAH, DAS, etc.)
9. Line 132-133 – "as previously…" missing a word
10. Supplementary Table 2 is set up in an unintuitive way, where columns 2 and 3 (means for each model) don't seem directly related to columns 4-7 (pairwise comparisons). This could be split into 2 tables, or organized differently
11. Supplementary Table 3 – DAH always has a plurality of "best-model count[s]", but in terms of AIC, BIC, and logL, only about half of the HD cells are better fit by the DAH model compared to GA or DAS. Does that mean a sizeable number of HD cells are better tuned to GA or DAS compared to DAH? Is the population heterogeneous in its tuning preferences? Pearson's R shows a higher number of cells most sensitive to DAH, but the authors indicated that Pearson's R was only included for interpretablity (Line 412)
12. Supplementary Figure 4 – There appears to be a very minor distinction (if any) between DAH and other models in terms of Rayleigh vector length, but a large difference in terms of kappa. Since kappa and Rayleigh vector length measure something similar (concentration/sharpness of distribution) why don't these results seem to match? Moreover, the Rayleigh vector lengths do not seem to differ significantly in these scatter plots (they lie largely along the diagonal) despite the differences being highly significant in Supplementary Table 2. Please clarify.
13. It's unclear if different numbers of parameters were actually estimated for each of the model fits (line 410 says "$k \in \{2,3\}$" but the authors should be more explicit with exactly which free parameters differs between models), as otherwise there is no reason to use AIC, BIC, and logL, as they will all give the exact same model comparison results (suggested by identical results Supplementary Table 3).
14. There are several places in the manuscript where visual inspection was used to determine results rather than statistics, such as lines 190-199 where the authors determined visually that the HD cells did not have true place tuning. This is an important question that deserves a more thorough analysis. One option would be to use the maximum likelihood technique from the paper they reference in that paragraph (Cacucci et al., 2004) to disambiguate place from HD tuning.
15. Line 241 – please define "poorlady microdrive"
16. Line 310 – Supp Fig 1 should be Supp Fig 2
17. Line 342-355 – spatial information is discussed in the methods but not in the main text – what was it used for? The authors again cite the Cacucci 2004 paper with refence to place x direction tuning, but they should also use the maximum likelihood method from that paper if they really want to distinguish place from direction tuning among their HD cells
18. Line 442 – says "ref" but there is no actual reference included

Reviewer #3

(Remarks to the Author)
Williams et al. set out to elucidate which of several competing models, particularly two "dual axis" models, might best explain updating of allocentric head direction when rodents travel in 3D space (a hemisphere). They find evidence that tuning parameters of cells best align to a "dual axis" updating mechanism using the head axis (tilt of the dorsal-ventral plane of the head in space). This paper is well written, the plan is well thought out to address this complex problem and while the results are quite interesting, I am not convinced that the interpretation is fully consistent with the data. The data do provide a compelling case that a dual axis – head axis (DAH) mechanism leads to the best tuning curves but the data does not appear to rule out the possibility that the other mechanisms that were considered are not also involved. In other words, it remains possible, based on the data presented here, that multiple updating mechanisms are involved. This was not considered in interpreting the data and should be.

Specifically, while the fit of the data to the dual axis – head axis model is best, both the gravity axis (GA) and also the dual axis – surface axis (DAS) models also appear to fit the data very well. If the data were approached in a different way, would the conclusion be the same? For example, if general linear models were used to fit the data to the hypotheses, would this suggest that the data fit each of these hypotheses very well? This is my only major concern.

Minor:
1. Label for panel c is missing on Figure 2.
2. AIC and BIC approaches (Fig 3d) need some explanation in the main text. It is not appropriate to refer to these acronyms only and say see online methods for a description of these methods.

Version 1:

Reviewer comments:

Reviewer #1

(Remarks to the Author)
I thank the authors for their responses, which have addressed my remarks.

I would like to clarify my earlier comment regarding the definition of the GA rule (line 78). In the original version, I understood this rule to imply that azimuth would be updated only via rotations about a gravity-aligned axis. This interpretation was logical, since the other rules are based on azimuth being updated through rotations about specific axes. The revised version is now clearer on this point.

I have one minor comment concerning lines 326–329 in the Discussion. If the HD signal is planar, that is, strictly one-dimensional, would this not also imply that HD cells fail to encode pitch? In other words, would they not “sacrifice” two degrees of freedom rather than one? If so, why is only roll discussed here?

Finally, a small correction regarding reference 38: Yakusheva et al. did not record cells encoding the earth-horizontal components of rotation. They exclusively reported cells sensitive to linear motion and only postulated the existence of a mechanism encoding earth-horizontal rotational components, which was later identified by Laurens et al. (Neuron 2013).

Reviewer #2

(Remarks to the Author)
This is a revised manuscript which demonstrates that HD cells follow a dual-axis updating rule while rats locomote on a hemisphere, with the best-fitting combination of axes being the animal’s dorso-ventral head axis along with the gravity axis (DAH model). The authors’ revisions in response to my previous criticisms are overall sufficient, and especially the addition of more explanatory/motivating text and a devoted discussion section have significantly improved the paper.

I do have one remaining critique regarding the newly added discussion, which may come down to a misunderstanding on my part, but could be clarified further for other readers. In the section “Implications of a planar HD signal”, the authors state that (line 246) “in the case of rats, the missing signal is the amount of roll around the nose axis, which does not modulate the signal for rotations of at least +/- 90 deg (Shinder and Taube, 2019)”. While it may be true that, when the rat’s nose axis is parallel with the ground plane, roll about the nose axis will cause neither a change in local azimuth nor continuous rotation of the head axis about the gravity axis, at other orientations (e.g., when the nose axis points upward) roll about the nose axis will cause the head axis to rotate about the gravity axis, which should modulate the signal according to the DAH rule (and in the case of the nose axis pointing straight upward, pitch would be expected to lack a modulatory effect). The Shinder study does state that “HD cells are primarily sensitive to yaw rotations with limited sensitivity to pitch and roll up to +/- 90deg” with a proposed reasoning that the posterior and superior canals are less effective than the horizontal canals for updating the HD signal, but that study does not identify roll in particular as a “missing signal” as the authors suggest here. The authors should clarify or remove this statement.

Reviewer #3

(Remarks to the Author)
The authors have addressed my original concerns and I have no further concerns.

Open Access This Peer Review File is licensed under a Creative Commons Attribution 4.0 International License, which permits use, sharing, adaptation, distribution and reproduction in any medium or format, as long as you give appropriate credit to the original author(s) and the source, provide a link to the Creative Commons license, and indicate if changes were made.
In cases where reviewers are anonymous, credit should be given to 'Anonymous Referee' and the source.
The images or other third party material in this Peer Review File are included in the article's Creative Commons license, unless indicated otherwise in a credit line to the material. If material is not included in the article's Creative Commons license and your intended use is not permitted by statutory regulation or exceeds the permitted use, you will need to obtain permission directly from the copyright holder.
To view a copy of this license, visit https://creativecommons.org/licenses/by/4.0/

Reviewer #1:

The data analysis is rigorous and follows state-of-the-art methods. Overall, this is a methodologically robust, highly novel, and convincing study that addresses an important question about the information encoded by HDCs during natural 3D motion. The findings are highly relevant to the broader field of spatial navigation.

We thank the reviewer for this positive assessment

Description of the GA rule (line 78): The description of the GA rule in line 78 is unclear and potentially inconsistent with the definition in line 414. Based on the definition in line 78, head rotation around an axis perpendicular to gravity would not update HDC. Yet, consider the following example: first, imagine that the head is vertical and define a vector u aligned with the nose. Second, pitch the nose up while keeping the vector u fixed in an earth reference frame. Third, rotate the head around u. Since u is orthogonal to the gravity axis, a would not be updated according to the definition in line 78. On the other hand, according to the definition in line 414, a would vary since the projection of the nose vector onto the earth-horizontal plane would change.

It wasn't quite clear what description the reviewer was referring to in line 78, which says only "a purely horizontally referenced updating rule" – however, assuming this phrase is what they mean, then this is not inconsistent with the description in line 414. This says "[the GA rule] involved projecting the nose vector onto the horizontal plane and computing azimuth (head direction in the horizontal plane) in the standard way." In the scenario the reviewer describes, rotating around u (a horizontal axis) changes the angle of the projection of the nose vector onto the horizontal plane (the horizontal reference frame alluded to in L78; see figure below for why this is so), and so *α* would in fact update. There may have been some confusion about what "purely horizontally referenced" means, so we have clarified as: "purely horizontally referenced updating rule (GA) describing global azimuth only".

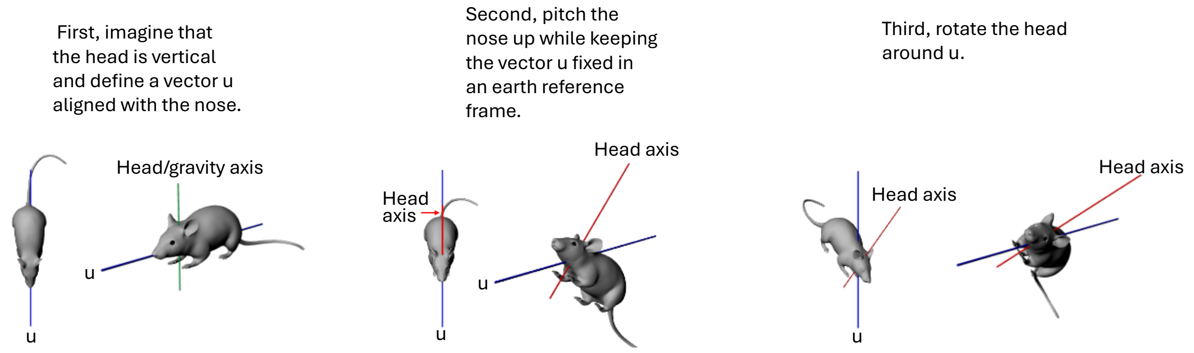

- Figure 3: For clarity, does the R value correspond to the correlation between the experimentally measured firing rate of the cell (as a function of time) and the firing rate predicted by the respective model, as defined in line 410?

It corresponds to firing rate as a function of alpha rather than time (that is, the tuning curve histogram) – this has been clarified with an addition to Supp. Fig. 3 that shows the analysis pipeline. The sentence in line 410 has been moved to section 6 of that paragraph and the legend in the new Fig. 3 clarified:

> Pairwise comparisons between GA and DAS, DAH and HA models, plus a direct DAS-DAH comparison, for the four parameters from the tuning curve histograms. The model fit represents the Pearson's R of the von Mises function fitted to the re-framed tuning curves. Solid black line shows y = x; dotted black line shows a linear fit to the actual data (ADN and POS combined). Note that the DAH model consistently outperforms the others (points lie above the y = x line) for almost all cells.

We did also try fitting the models directly to the time-series data but the fits were poorer and also took a very long time to calculate.

- Equation 2: The authors seem to imply that αHH and αHG are two variables represented independently in the brain. Is this what they actually mean? Consider the following examples:

(1) Starting from the upright head position (both α = 0), the head rotates 360° in yaw. This corresponds to αHH = 360° and αHG = 0.

(2) Starting from the same position, the head pitches up until the nose is vertical, then rotates 360° around the gravity axis, and finally pitches back to horizontal. This corresponds to αHH = 0 and αHG = 360°.

In both cases, the initial and final head orientations are identical. However, if αHH and αHG are represented separately by the HD system, then the overall neuronal activity at the final position would differ between the two trajectories. What is the authors' interpretation of this? Note that I am not questioning the validity of the dual updating rule itself, but rather whether the HDC system represents two distinct forms of "azimuth."

In the example given, 0 and 360 deg. would be, neurally, the same place on the ring attractor, so there is no conflict here. It isn't yet clear whether the two axes are represented distinctly somewhere in the brain and indeed this is an important question for future research. The semicircular canals (for head rotation) and otolith organ signals (for gravity) converge early in the pathway, at the vestibular nucleus in the brainstem, so it is possible that the combined rotation is calculated almost from the beginning before being sent to the ring attractor. However, it's also possible that the attractor network is where these signals are combined. A speculation about how and where these signals might be computed has been added to the discussion.

- Line 222: Related to the point above, the authors write that "the added role of GA azimuth requires extension of standard ring attractor models." Alternatively, one could propose that HA and GA rotation velocities are computed upstream of the ring attractor and that the sum of these signals is provided to the attractor. This would comply with the dual-axis updating rule without requiring an extension of the standard model.

This is true, if one considers the model only to include the ring itself and not the information content of its inputs. We prefer to take the broader view, in order to promote future research interest in understanding this information. It should be noted that the input doesn't have to be a velocity – as we now describe in a new Discussion section, the inputs could also derive from static landmarks that reset the signal.

- Line 175: As a side result, the authors report no tuning to elevation. However, the range of elevation sampled in their study appears rather narrow (Fig. S5). It is conceivable that some tuning to elevation (or other forms of 3D tuning) exists but cannot be observed given the limited elevation range sampled. I would encourage the authors to take that limitation into account.

It is true that the range of elevations was rather small: we have now said:

> Neither visual inspection nor statistical analysis (not reported) found evidence of a systematic elevation-rate relationship, although this cannot be ruled out given the limited pitch sampling. However the observation is consistent with previous work by Shinder and Taube [11].

Reviewer #2:

Results indicate fairly convincingly that the DAH model provides the best fit for most cells.

We are pleased that the reviewer finds the results convincing

1. The main question of whether the local axis is referenced to the environment surface or the head itself is important, but not clearly motivated by the introductory text, other than mentioning that previous studies "did not address" it (line 66) and that "either is plausible" (line 67). Are there any reasons to expect one hypothesis over the other (DAH or DAS)? Does anything from the cited previous studies (or others – see point 3) indicate that one local axis is more likely? As HD cells have been demonstrated to rely on vestibular inputs, is there reason to believe they wouldn't be referenced to the animal's head itself (which already contains vestibular organs that detect head turns and gravity orientation)? Is there a potential biological basis for HD cells being somehow referenced to the plane tangent to the local surface? More thorough motivation of the experiment and reasoning behind each hypothesis is needed.

This was actually our subsidiary hypothesis, with our main one being operation of a DA rule, but we take the point that we could have motivated this more clearly. We think

either reference frame is possible, given the known importance of allocentric (environment-based) reference frames for place cells and grid cells. Previous work with overhead cameras has always referenced the signal to the horizontal plane, so whether it is the head or the plane itself has not been experimentally disambiguated. We are not the only investigators to have considered that the plane itself might be paramount: Shinder and Taube (2018) wrote:

> One possibility, referred to as the rotational plane hypothesis, is that directions are represented in a manner that reflects the plane the animal is currently locomoting in. Thus the walls represent extensions of the floor and head rotation in the plane of locomotion is aligned to a PFD in that plane regardless of the animal's orientation relative to gravity. The second possibility for how HD cells encode direction is that directional headings are referenced to gravity. In this case, the firing of HD cells depends on how the three semicircular canals are oriented relative to gravity. In this view, angular movements in the horizontal plane are detected by different vestibular canals depending on their alignment with gravity.

These authors, like the reviewer (and indeed ourselves) considered that the gravity reference frame was more plausible, but it remained an untested assumption until now. To clarify this issue we have elaborated our introductory text..

Is there a potential biological basis for HD cells being somehow referenced to the plane tangent to the local surface?

Not that we yet know of, but hypothetically, grid cells might serve to provide a reference plane.

2. Related to point 1, there is also very little discussion of the results and their implications for spatial coding in the brain as well as behavior/navigation. For example, the authors conclude the main text by stating "How the brain processes these reference frames, and how and where they interact, remains to be elucidated" (Line 223-225). But there has been a sizable amount of experimental and theoretical work devoted to answering this question (see Laurens and Angelaki 2018 for review), and at least some of this work should be discussed to properly contextualize the results of the current study.

With the expanded word count available in this version we have now developed the introduction and added a separate Discussion section that explores these issues in detail.

Additionally, the authors should mention if there are any implications of their results for spatial coding downstream of the HD circuit. For example, entorhinal grid cells and hippocampal place cells are typically considered to represent allocentric locations on a

particular surface. Further, grid cells have been shown to depend on anterior thalamus functon(Winter et al., 2015) and place cells and HD cells are strongly coupled in their orientation preferences (Knierim et al., 1995), suggesting that these systems interact. Since the current study shows that HD cell reference axis is egocentric, is there some transformation that needs to occur for the egocentric HD signal to properly interact with downstream allocentric grid and place signals (among other allocentric cell types)? Would the distinction between surface-referenced and head-referenced tuning in the ADN/PoS be expected to have specific effects on downstream neuronal firing properties, e.g. entorhinal or hippocampal cells, or on behavior in general? Overall a dedicated Discussion section would be welcome.

It is only partially true that the "HD cell reference axis is egocentric," as this is just one of its two axes, the second (GA) being allocentric. What we have shown is that by summing signals in the two reference frames, the egocentric rotation of the head is straightforwardly converted to allocentric coordinates (with some slight deviations (now shown in the new Fig. 1e). However we appreciate the suggestion to speculate on the implications of HD coding for downstream spatial neurons and we have now discussed this in the new Discussion section.

3. Mentions of several directly relevant studies are missing; for example, the paper mentions (Page et al., 2018) and (Laurens and Angelaki 2018) as recent evidence for the DA rule, but misses (Angelaki et al., Nat Comms, 2020), which showed evidence for the DA rule in mice on an orientable platform, and (LaChance et al., J Neurosci, 2020), which showed evidence for the DA rule in rats climbing on different faces of a cube.

A discussion of these studies has been added to the expanded Introduction.

The (Angelaki et al., 2020) study is particularly relevant to the discussion of elevation tuning, which was observed in that study but not in the current study, which should be discussed more thoroughly.

This has been added to the Discussion.

Many of the concepts critical to this paper were also explored in (Shinder and Taube, J Neurophys, 2018) which investigated HD tuning in head-fixed rats subjected to a variety of passive 3D rotations, and also concluded that a dual-axis rule was responsible for HD cell firing preferences (though they suggested that the local axis was primarily visual). The Shinder and Taube study may also be relevant because HD cells fired normally during passive 3D rotations despite the lack of a traditionally defined 'local surface' (the animals were head-fixed inside a tube).

These have now been explored in the Discussion.

Minor points

1. Line 32 – "exploring a hemisphere", and Line 70 – "exploring a hemispherical arena" – need make clearer that the animals are crawling on the surface of the hemisphere instead of moving inside it (unlike the Grieves lattice maze, for example)

We have changed to "hemispherical surface" throughout.

2. Line 50-51 – "However, HD cells do maintain a stable PFD on a sphere (Fig. 1b)…." The authors cannot make this definitive statement based solely on a single path/spike plot from one HD cell, they need to demonstrate it statistically or at least show more examples

We have clarified in the text that this figure just shows an example – the full set of 436 cells with good directional tuning throughout recording sessions supports the general claim that tuning is not lost on a highly curved surface (see 40 examples in the new Supp. Fig. 5 – the GA plots show the good tuning in the horizontal reference frame).

3. Fig. 1b – why does the side view only show a path with no cell firing, while the top views show cell firing but don't include a path alone?

These figures were intended to make different points: the path plot is to show the exploration patterns of the animals, which deviate most from normal at the sides of the sphere where the surface Is steepest. The view from the top of the spike-plots was to illustrate the difference in directional tuning between HD and non-HD cells and how tuning was uniform across the apparatus for the HD cell.

It could also be useful to see some occupancy distributions for locations on the top/sides of the sphere, since it seems like the animals rarely walked on the steep sides of the sphere (where the distinctions between the different models should be greatest)

The animals did spend more time on the top of the sphere than the sides, but did explore the sides as well. Additional examples have been added to the Supplementary figures (Supp. Fig. 3a). There are indeed sampling inhomogeneities which is why traditional method of tuning curve determination could not be applied.

4. Related to above point, the authors should include more examples of these path/spike plots for different cells so readers can better assess behavior and cell firing

Additional examples have been added as Supp. Fig. 3b.

5. Fig. 1d – axis labels are overlapping which makes them hard to read and interpret

This has been fixed.

6. Fig. 1e – the GA and DAH examples are clearly the same cell as mentioned in the legend (same vector locations), but why does the DAS example appear to be from a different cell/recording?

This was an accidental duplication, as all three should have been different (because the different reference frames lead to different binning). However on reflection we have decided this is not the most valid way to make this point, as the vectors were generated using directional dwell bins that were incompletely sampled (hence, as mentioned above, our adoption of a different analysis method for the complete data set), and so we have replaced this figure part with a schematic.

7. Fig. 1f – it would be useful to see the actual divergence between local PFDs and global PFD at different locations on the sphere (e.g., what is the degree or radian difference represented by the hottest color?). Do the actual HD cells show differences of a similar pattern/magnitude, either individually or as a population (an empirical version of this theoretical plot would be nice to see)? This may not be possible given the limited sampling on the sides of the sphere.

This unfortunately isn't possible because we didn't directly measure local PFD: we inferred it by taking the global PFD and then making adjustments based on the DA rule. Thus, the divergence would follow the theoretical divergence distribution as a matter of necessity.

8. The authors should include more tuning curve examples in addition to the one shown in Fig. 2a. There are 40 example HA cells in Supplementary Fig. 6 which is the least common cell type, so they should be able to show more examples of the more common cell types (DAH, DAS, etc.)

We have now added (new Supp. Fig. 5) 40 example cells chosen from across the whole data set (approx. every 10th cell). Note that we have now removed the HA analysis, as our revised model selection process, based on the best Pearson's R model fit, found no cells for which the HA reference frame was the best model, and inspection of the additional scatterplots (now Fig. 3) showed no evidence of a cluster of HA-tuned cells. We believe these cells, previously selected using peak rate alone, just reflected noisy data.

9. Line 132-133 – "as previously..." missing a word

Corrected

10. Supplementary Table 2 is set up in an unintuitive way, where columns 2 and 3 (means for each model) don't seem directly related to columns 4-7 (pairwise comparisons). This could be split into 2 tables, or organized differently

The tables have been reorganized and simplified.

11. Supplementary Table 3 – DAH always has a plurality of "best-model count[s]", but in terms of AIC, BIC, and logL, only about half of the HD cells are better fit by the DAH model compared to GA or DAS. Does that mean a sizeable number of HD cells are better tuned to GA or DAS compared to DAH? Is the population heterogeneous in its

tuning preferences? Pearson's R shows a higher number of cells most sensitive to DAH, but the authors indicated that Pearson's R was only included for interpretablity (Line 412)

This is a valid point, and we revisited the analysis (prompted by both this comment and the similar point below) and have decided to remove it and restrict ourselves to the analysis of the tuning curve parameters. These are now shown in detail in our new Fig. 3 where it can be seen that there is no meaningful heterogeneity: very few cells fall below the y = x line when DAH (y axis) is compared with the other models, and these do not seem to form a separate category but rather just reflect noise in the data. We thus do not think this is a mixed population.

For the same reason we have, as mentioned above, reconsidered the analysis of the "HA" cells and have concluded that these likely reflect noise also, and so have removed the analysis and discussion that speculated about these.

12. Supplementary Figure 4 – There appears to be a very minor distinction (if any) between DAH and other models in terms of Rayleigh vector length, but a large difference in terms of kappa. Since kappa and Rayleigh vector length measure something similar (concentration/sharpness of distribution) why don't these results seem to match? Moreover, the Rayleigh vector lengths do not seem to differ significantly in these scatter plots (they lie largely along the diagonal) despite the differences being highly significant in Supplementary Table 2. Please clarify.

This was a graphical error on our part, and thank you for catching it. We had overlooked that for some reason a subset of the solid black x=y lines failed to export from the svg to the jpeg. That has been rectified and it is now more apparent in the figure (now Fig. 3 in the main text) that the DAH model is superior for both kappa and Rayleigh vector measures.

13. It's unclear if different numbers of parameters were actually estimated for each of the model fits (line 410 says "k∈{2,3}" but the authors should be more explicit with exactly which free parameters differs between models), as otherwise there is no reason to use AIC, BIC, and logL, as they will all give the exact same model comparison results (suggested by identical results Supplementary Table 3).

We agree and have removed this analysis, as mentioned above.

14. There are several places in the manuscript where visual inspection was used to determine results rather than statistics, such as lines 190-199 where the authors determined visually that the HD cells did not have true place tuning. This is an important question that deserves a more thorough analysis. One option would be to use the maximum likelihood technique from the paper they reference in that paragraph (Cacucci et al., 2004) to disambiguate place from HD tuning.

We agree this would be a valid approach of we wished to argue that there *is* place tuning, but since visual inspection, corrected for sampling bias, did not convincingly reveal place coding, the analysis would be trying to prove a negative result which we don't feel is useful in this context. In light of the reviewer's comments we have decided it would be better to remove this observation, which is a side distraction from the main issue.

15. Line 241 – please define "poorlady microdrive"

This is an informal term and has been removed.

16. Line 310 – Supp Fig 1 should be Supp Fig 2

Thank you – fixed.

17. Line 342-355 – spatial information is discussed in the methods but not in the main text – what was it used for? The authors again cite the Cacucci 2004 paper with refence to place x direction tuning, but they should also use the maximum likelihood method from that paper if they really want to distinguish place from direction tuning among their HD cells

As mentioned above, we have removed the place analysis.

18. Line 442 – says "ref" but there is no actual reference included

Fixed.

Reviewer #3:

This paper is well written, the plan is well thought out to address this complex problem and while the results are quite interesting…

We are pleased the reviewer considers these findings interesting

… *I am not convinced that the interpretation is fully consistent with the data. The data do provide a compelling case that a dual axis – head axis (DAH) mechanism leads to the best tuning curves*

We are also pleased that this aspect is compelling

… *but the data does not appear to rule out the possibility that the other mechanisms that were considered are not also involved. In other words, it remains possible, based on the data presented here, that multiple updating mechanisms are involved. This was not considered in interpreting the data and should be.*

We agree that there may be other updating mechanisms or (relatedly) other factors affecting HD cell firing rate as well: arousal, heterogeneous inputs from visual landmarks etc. Given this, the underlying tuning curves could in principle be even narrower, and with the added information the model fit could be further improved (it fell

far short of 1.0). However, it is beyond the scope of the study to do further experiments to explore other factors, and we would also be testing a different hypothesis. In terms of existing data, we have only the head directions, positions and firing rates available for analysis. Within the constraints of those data we did explore whether spatial or pitch information might contribute to some of the cells' activity, but could not convince ourselves of this.

Specifically, while the fit of the data to the dual axis – head axis model is best, both the gravity axis (GA) and also the dual axis – surface axis (DAS) models also appear to fit the data very well. If the data were approached in a different way, would the conclusion be the same? For example, if general linear models were used to fit the data to the hypotheses, would this suggest that the data fit each of these hypotheses very well? This is my only major concern.

The GLM in this case is the von Mises function that mathematically describes the tuning curves under each of the transformations, so we think the reviewer is suggesting that while the DAH models fit best, the fact that DAS and GA provide reasonable fits as well suggests there may be other models that are even better. We agree there is room for improvement in the model fits: while DAH is superior to the traditional horizontal reference frame, we cannot rule out the possibility that there are other factors that could be added to improve the model further (related to the point above).

1. Label for panel c is missing on Figure 2.

Thank you for the catch - fixed

2. AIC and BIC approaches (Fig 3d) need some explanation in the main text. It is not appropriate to refer to these acronyms only and say see online methods for a description of these methods.

We have removed this analysis based on Reviewer 2's comments.

Reviewer #1

I have one minor comment concerning lines 326–329 in the Discussion. If the HD signal is planar, that is, strictly one-dimensional, would this not also imply that HD cells fail to encode pitch? In other words, would they not "sacrifice" two degrees of freedom rather than one? If so, why is only roll discussed here?

This is a good point and we have amended to "The DA scheme yields a planar signal, as contrasted with a fully volumetric 3D signal which would encode the nose vector in all three rotational planes. This lower dimensional representational format is therefore unable to signal every possible head orientation in 3D space. Why would evolution sacrifice these other degrees of freedom?"

Finally, a small correction regarding reference 38: Yakusheva et al. did not record cells encoding the earth-horizontal components of rotation. They exclusively reported cells sensitive to linear motion and only postulated the existence of a mechanism encoding earth-horizontal rotational components, which was later identified by Laurens et al. (Neuron 2013).

We have replaced the Yakusheva et al. reference with the newer one suggested.

Reviewer #2

I do have one remaining critique regarding the newly added discussion, which may come down to a misunderstanding on my part, but could be clarified further for other readers. In the section "Implications of a planar HD signal", the authors state that (line 246) "in the case of rats, the missing signal is the amount of roll around the nose axis, which does not modulate the signal for rotations of at least +/- 90 deg (Shinder and Taube, 2019)". While it may be true that, when the rat's nose axis is parallel with the ground plane, roll about the nose axis will cause neither a change in local azimuth nor continuous rotation of the head axis about the gravity axis, at other orientations (e.g., when the nose axis points upward) roll about the nose axis will cause the head axis to rotate about the gravity axis, which should modulate the signal according to the DAH rule (and in the case of the nose axis pointing straight upward, pitch would be expected to lack a modulatory effect). The Shinder study does state that "HD cells are primarily sensitive to yaw rotations with limited sensitivity to pitch and roll up to +/- 90deg" with a proposed reasoning that the posterior and superior canals are less effective than the horizontal canals for updating the HD signal, but that study does not identify roll in particular as a "missing signal" as the authors suggest here. The authors should clarify or remove this statement.

This is the same concern as expressed by reviewer 1, and as mentioned above, we have removed this statement as recommended.
